# Myocardial impulse propagation is impaired in right ventricular tissue of Zucker Diabetic Fatty (ZDF) rats

**DOI:** 10.1186/1475-2840-12-19

**Published:** 2013-01-17

**Authors:** Kristine Boisen Olsen, Lene Nygaard Axelsen, Thomas Hartig Braunstein, Charlotte Mehlin Sørensen, Claus B Andersen, Thorkil Ploug, Niels-Henrik Holstein-Rathlou, Morten Schak Nielsen

**Affiliations:** 1 The Danish National Research Foundation Centre for Cardiac Arrhythmia and Department of Biomedical Sciences, Faculty of Health Sciences, University of Copenhagen, Blegdamsvej 3, Copenhagen DK-2200, Denmark; 2 Department of Biomedical Sciences, Faculty of Health Sciences, University of Copenhagen, Copenhagen, Denmark; 3 Department of Pathology, Rigshospitalet, University of Copenhagen, Copenhagen, Denmark

**Keywords:** Diabetic cardiomyopathy, Arrhythmia, Lipotoxicity, Conduction velocity, Gap junctions, Type 2 diabetes, Zucker Diabetic Fatty (ZDF) rats

## Abstract

**Background:**

Diabetes increases the risk of cardiovascular complications including arrhythmias, but the underlying mechanisms remain to be established. Decreased conduction velocity (CV), which is an independent risk factor for re-entry arrhythmias, is present in models with streptozotocin (STZ) induced type 1 diabetes. Whether CV is also disturbed in models of type 2 diabetes is currently unknown.

**Methods:**

We used Zucker Diabetic Fatty (ZDF) rats, as a model of type 2 diabetes, and their lean controls Zucker Diabetic Lean (ZDL) rats to investigate CV and its response to the anti-arrhythmic peptide analogue AAP10. Gap junction remodeling was examined by immunofluorescence and western blotting. Cardiac histomorphometry was examined by Masson`s Trichrome staining and intracellular lipid accumulation was analyzed by Bodipy staining.

**Results:**

CV was significantly slower in ZDF rats (56±1.9 cm/s) compared to non-diabetic controls (ZDL, 66±1.6 cm/s), but AAP10 did not affect CV in either group. The total amount of Connexin43 (C×43) was identical between ZDF and ZDL rats, but the amount of lateralized C×43 was significantly increased in ZDF rats (42±12 %) compared to ZDL rats (30±8%), p<0.04. Judged by electrophoretic mobility, C×43 phosphorylation was unchanged between ZDF and ZDL rats. Also, no differences in cardiomyocyte size or histomorphometry including fibrosis were observed between groups, but the volume of intracellular lipid droplets was 4.2 times higher in ZDF compared to ZDL rats (p<0.01).

**Conclusion:**

CV is reduced in type 2 diabetic ZDF rats. The CV disturbance may be partly explained by increased lateralization of C×43, but other factors are likely also involved. Our data indicates that lipotoxicity potentially may play a role in development of conduction disturbances and arrhythmias in type 2 diabetes.

## Introduction

Diabetes is a major risk factor for sudden cardiac death and ventricular tachy-arrhythmias are suspected to be the predominant mechanism [1]. The prevalence of ventricular arrhythmias is increased in patients with diabetes and although ischemia is suspected to be an important trigger, the increased risk is independent of co-morbidities like coronary heart disease or heart failure [2,3].

The electrocardiogram (ECG) of the diabetic heart is often characterized by a prolonged QT interval, reflecting an increase in action potential duration. In support of this, both the IKto- and delayed rectifier currents are reduced in ventricular cardiomyocytes from type 1 diabetic rats resulting in action potential prolongation (For review see [4]). Furthermore, heterogeneous potassium current remodeling increases the dispersion of repolarization and thereby increases the risk of arrhythmia.

Intrinsic differences in repolarization properties exist even in the healthy heart and can exacerbate during conditions of gap junction uncoupling, where local differences are no longer smoothened [5,6]. Reduced coupling not only increases the repolarization gradients and the risk of conduction block, it also reduces conduction velocity (CV), which increases the risk that the heart can harbor a reentrant circuit [7].

CV is determined by excitability, internal electrical resistance and cardiac structure. The internal electrical resistance can be viewed as two resistors in series; the resistance of the cell cytoplasm, which is determined by the cellular composition, e.g. the amount of intracellular organelles and non-conducting material like lipid droplets, and the resistance at the cell-cell junctions [8]. In the ventricles of the heart, the later is determined by the expression, localization and posttranslational regulation of gap junctions composed of the gap junction protein connexin43 (C×43) (for recent review see [9]). More specifically, C×43 expression, localization, and phosphorylation [10-12], as well as changes in pH [13], [Ca^2+^[13], lipid metabolites [14,15] and phosphatidylinositol-bisphosphate 2 (PIP2) levels [16] have been shown to affect the resistance of C×43 gap junctional channels. In addition, studies suggest that C×43 undergo remodeling in type 1 diabetes, however, data are not consistent, ranging between reduced [17,18], unchanged [19,20] and increased [20,21] C×43 expression. Some studies link type 1 diabetes to increased lateralization of C×43 [19,20], and type 1 diabetes also affects phosphorylation of C×43 judged by electrophoretic mobility [17,20] or phospho-specific antibodies [21].

All present knowledge of the effect of diabetes on CV and gap junction remodeling comes from studies on rats with streptozotocin (STZ) induced type 1 diabetes. STZ is a toxic chemical, which induces type 1 diabetes by destroying pancreatic beta-cells, thereby preventing insulin secretion. Whether the gap junction and conduction changes observed in STZ induced type 1 diabetic rats are also present in models of type 2 diabetes remain to be established. Therefore, we set out to evaluate CV as well as C×43 expression, localization and phosphorylation along with lipid accumulation and histological changes in Zucker Diabetic Fatty (ZDF) rats. ZDF rats are a well described model of type 2 diabetes, induced by a mutation in the leptin receptor. ZDF rats develop obesity, hyperglycemia, hyperlipidemia and overt diabetes (For review see [22]). Along with these systemic alterations, ZDFs also demonstrate cardiac changes such as altered myocyte contraction and Ca^2+^ signaling at 9 to 13 weeks of age [23], mild diastolic dysfunction and increased collagen content at 45 weeks of age [24], as well as increased cardiac levels of triacylglycerol at 6 and 7 weeks of age [25,26]. Alterations in the ECG with the onset of diabetes in ZDF rats have also been demonstrated [27]. These alterations include widening of the QRS intervals, which may indicate decreased ventricular CV.

In this study, we find that ZDF rats have a decreased CV in the right ventricle compared to ZDL rats. ZDF rats had unaltered C×43 expression and phosphorylation, but an increase in the content of lateralized C×43 was observed. In addition, we show that there is no effect of the anti-arrhythmic peptide analogue AAP10, which is otherwise known to preserve gap junction coupling and improve CV during other pathophysiological conditions such as ischemia [28,29]. At the same time, we find no histomorphometric changes in the ZDF hearts, but they do show significant intramyocardial lipid accumulation compared to their controls.

## Methods and Materials

### Animals

All animal studies were performed according to the *Guide for the Care and Use of Laboratory Animals*, published by the United States National Institutes of Health (NIH publication No. 85–23, revised 1996), and approved by the Animal Experiments Inspectorate of the Danish Ministry of Justice. Male Zucker Diabetic Fatty rats (n=16) and male Zucker Lean rats (n=21) (Charles River Laboratories, Margate, England) between 12–17 weeks of age were used. Animals were housed on a 12-hour dark/12-hour light cycle. They were fed a Purina 5008® rat chow containing 23% protein, 6.5% fat, 58.5% carbohydrates, 4% fiber and 8% ash and they received tap water ad libitum.

### Assessment of biochemical parameters and blood pressure

Rats were anaesthetized with 5% Isoflurane (Baxter, Allerød, Denmark) delivered in 65% N_2_ and 35% O_2_. A tracheotomy was performed, and the rat was placed on a servo-controlled heating table to maintain a constant body temperature of 37°C. A small-animal ventilator ensured that the rat was ventilated by a tidal volume of 2.0–2.7 ml at 60 breaths/min and anesthesia was reduced to 2% isoflurane. A polyethylene catheter (PE-50) was placed in the right carotid artery for continuous measurement of mean arterial blood pressure (MAP) by a Statham P23-dB pressure transducer (Gould, Oxnard, CA). Blood pressure was sampled for 5 min with a Powerlab/8SP system (AD instruments, Colorado Springs, CO, USA). Following the pressure measurement a blood sample was collected for determination of triglycerides, using a Reflotron (Roche, Hvidovre, Denmark).

### Electrophysiological and mechanical measurements

Following MAP measurements and blood collection, hearts were removed and immersed in warm Tyrode’s buffer of the following composition (mmol L^-1^): NaCl 136, KCl 4, MgCl2 0.8, CaCl2 1.8, HEPES 5, 2-(N-morpholino)ethanesulfonic acid (MES) 5, Glucose 10, pH 7.3. Subsequently, a tissue strip from the right ventricle was dissected and used for CV measurements as previously described [30]. In short, the tissue strip was superfused with oxygenated Tyrode’s buffer at a rate of 2 mL/min and the temperature was maintained at 37°C. The tissue was paced at 1 Hz, an impulse duration of 0.5 ms and voltage was set to two times the threshold value.

A length-tension curve was made for each tissue strip followed by adjustment of the muscle length to the level where developed tension was 50% of the maximal tension. Force was continuously recorded by a force-transducer. Changes in developed force and passive tension during the experiments were analyzed and calculated by custom written software. At the end of experiments, the length of the tissue strip was determined using a microscope with a calibrated ocular grid (Wild M38, Heerbrugg, Schweiz) and the tissue was weighed. Cross-sectional area was calculated by dividing muscle mass (volume) by muscle length and a density of 1,063 mg/mm^3^ (cross-sectional area (mm^2^) = volume (mm^3^)/length (mm)). Developed force and resting tension were normalized for cross sectional area.

CV was measured with two microelectrodes (Platinium/Iridium (PI20030.5B10), Micro Probe Inc., Gaithersburg, USA) placed on the longitudinal axis of the tissue strip. The distance between the two microelectrodes was measured with the calibrated ocular grid in the microscope. The distance between the two microelectrodes was 1.2-2.2 mm and distance between the stimulation electrode and the first microelectrode was 1.7-3.4 mm. The extracellular field potentials and force were band pass filtered at 300 – 10,000 Hz and sampled at 10 kHz (Digidata 1322A, Axon Instruments, Union City, USA). Time of local activation under the first and second microelectrode was determined as the time of minimum dU/dt by the custom written software. CV was calculated as the inter-electrode distance divided by the inter-electrode delay.

### Experimental protocol

Tissue strips were allowed to stabilize for 20 minutes in all experiments. Subsequently, CV was measured for 40 minutes and in a subset of experiments, the tissue strips were treated with AAP10 (50 nmol/L) or vehicle (Milli-Q water) during the last 20 min of the measurements. At the end of the experiment, the tissue was frozen in liquid nitrogen and stored at −80°C for later western blot analysis and immunofluorescence.

### Western blot

Frozen tissue was crushed in liquid N_2_ and dissolved in cold RIPA buffer containing PMSF (1 mmol/L), sodium orthovanadate (1 mmol/L), Protease Inhibitor Cocktail and Phosphatase Inhibitor Cocktail 1 and 2 (Sigma-Aldrich, St. Louis, MO) in the concentrations recommended by the manufacturer. Samples were sonicated 3 times 10 sec on ice, centrifuged at 20,000 g at 4°C for 30 minutes and supernatants were analyzed for protein content (Bradford). Ten μg protein per lane was applied to a 7% NuPAGE gel (Invitrogen, Carlsbad, CA). After gel electrophoresis the protein was transferred to a nitrocellulose membrane, blocked and incubated in 5% skim milk with the primary antibody (C×43: C6219, 1:1500, Sigma-Aldrich, St. Louis, MO). After blocking, the membrane was incubated with a HRP-conjugated antibody (anti-rabbit; Thermo Fisher Scientific, Rockford, IL) followed by SuperSignal West Femto HRP-substrate (Thermo Fisher Scientific, Rockford, IL) and quantified in a UVP Epi Chem II Darkroom (UVP Inc., Upland, CA). In the outermost lanes on each side of each gel a sample of 20 μg rat ventricular heart tissue treated with alkaline phosphatase (P6774, Sigma-Aldrich, St. Louis, MO) was applied. This dephosphorylated C×43 represents the band corresponding to P0. Images were then quantified by measuring optical density in the P0 region as compared to the higher phosphorylated forms using LabWorks Analysis Software ver. 4.6 (UVP, Upland, CA). Each of the two regions in each lane was defined and measured three times and the average value was used. All measurements were performed in a blinded fashion.

### Immunofluorescence

Sections (10 μm) were cut from small samples of free ventricular wall frozen in liquid nitrogen using a LEICA CM3050S cryostat. The sections were subsequently fixed with 2% paraformaldehyde in PBS and permeabilized in PBS with 4% BSA and 0.2% Triton X-100. PBS with 4% BSA was used for all blocking steps. Tissue was incubated overnight with a primary C×43 antibody (C6219, Sigma-Aldrich, St. Louis, MO, 1:1000) and N-cadherin (C3865 Sigma-Aldrich, St. Louis, MO, 1:500) in PBS with 4% BSA. After washing, tissue was incubated with appropriate secondary ALEXA conjugated antibodies and counterstained with rhodamine-phalloidin for 45 min at room temperature (both Invitrogen, Carlsbad, CA). Tissue slides were washed in PBS and mounted in Prolong Gold mounting medium (Invitrogen) and imaged using a laser confocal microscope (Leica TCS SP2, Leica Microsystems, Wetzlar, Germany) using a 63X Water NA 1.2 objective.

*Quantification of total C×43 in ventricular tissue*: Random areas of longitudinally sectioned ventricular myocytes were chosen based on phalloidin staining only. The image plane emitting the highest C×43 signal was then selected for scanning. All images were recorded at 12 bit, 1024×1024 pixels (a field of 238.1×238.1 μm), 4× line average, while ensuring that no pixels were saturated. Image calculations were performed with ImageJ (Ver. 1.42d, National Institutes of Health, USA). Cell area was determined on the basis of phalloidin staining by setting a threshold for each image. For C×43 staining a global threshold enabling discrimination between background and C×43 signal was selected. The integrated intensity of the C×43 signal was then divided by the area of the cells to give the average C×43 per cell area signal intensity. For each rat 5 images were recorded and the average intensity of these 5 images was regarded as n=1. The total area of cells analyzed was 1.47*10^6^ μm^2^ for ZDF rats (n=7) and 1.17*10^6^ μm^2^ for ZDL rats (n=6). All images were recorded and quantified in a blinded fashion.

*Quantification of C×43 residing in intercalated discs:* Random areas of longitudinally sectioned ventricular myocytes were chosen and the settings for images were as described above. The intercalated disc was identified by N-cadherin staining. As C×43 and N-cadherin are localized in different parts of the intercalated disc and therefore do not perfectly colocalize, we defined the area of the intercalated disc as the area surrounding the N-cadherin staining. Images were thresholded individually and pixels above threshold were then “enlarged” by 10 pixels (corresponding to 2.3 μm) by use of the “Mask of Nearby Points” plugin for ImageJ (written by Mark Longair). Backgrounds of the corresponding C×43 images were individually thresholded and the integrated intensity of the pixels in the images was recorded. The enlarged mask from the N-cadherin image was then applied to the C×43 image and the integrated intensities of the total C×43 signal as well as the C×43 signal localized within the intercalated disc was recorded. Five images from each of the 14 animals (seven in each group) were recorded. All images were recorded and quantified in a blinded fashion.

### Quantification of intramyocardial lipid droplets

Small tissue strips from the free wall of the right ventricle was fixed with 2% Zamboni fixative and stored in 50% glycerol in PBS at −20°C until further analysis. Determination of intramyocardial lipid droplets was done by staining small “flakes” of the fixed tissue with Bodipy-493/503 (Invitrogen) as previously described [31] and subsequent analysis by confocal microscopy. In short, small flakes of the fixed tissue strip were transferred to PBS (pH 7.35) and incubated for 30 min with Bodipy-493/503 (2 mg/ml in PBS). After three washes with PBS for 10 min each, flakes were mounted in Vectashield (VectorLaboratories, Burlingame, CA) on a glass slide and analyzed by confocal microscopy. All recordings and postprocessing of the images were done in a blinded fashion.

*Quantification of lipid droplets:* Stacks of 14 images (covering 5.02 μm in the z-plane) with the x-y dimensions of 61.64 by 61.64 μm were recorded at six different randomly chosen locations in ventricular heart tissue from each rat with a Zeiss LSM 780 equipped with a 63x NA 1.4 oil lens. The resulting stacks were postprocessed by use of the free software package FiJi (formerly known as ImageJ) by the following procedure: The stack was smoothed and processed with a Laplacian differential filter. The images were then inverted and smoothed 4 times to level out differences in grey levels over the lipid droplets. Hereafter the stack was thresholded to a level where no structures of the background staining of the myocytes were visible and converted to 8 bits. The resulting stacks were then processed with the 3D Objects Counter (FiJi plugin), which calculated volumes of the thresholded lipid droplets. Sizes of the thresholded droplets were verified by visual comparison to the droplets in the original images. All quantifications were performed in a blinded fashion.

*Quantification of heart tissue volume:* The same stacks of images used for quantification of lipid droplets were smoothed twice and thresholded to a level showing tissue over background. Then the Multi measure plugin for FiJi was used to give percentages of tissue coverage in each image in the stack. The average percentage of tissue for a whole stack was then multiplied by the entire recorded volume (19073 μm^3^).

### Histomorphometric analysis

A part of free right ventricular wall was mounted in Tissue-Tek (Miles, Elkhart, IN) and frozen in isopentane, cooled to its freezing point in liquid nitrogen and stored at – 80°C till further analysis. Subsequently, samples were fixed in 4% formalin and processed for histological examination as previously described [32]. In short, 5 microns thick, paraffin-embedded sections were serially cut and stained with Masson`s Trichrome. For each case histomorphometric analysis was performed on a Masson's Trichrome stained slide at 100× magnification. From each slide, three microscopic fields were randomly selected. For each microscopic field, a grid with regular spaced points was used to count the number of points hitting cardiomyocytes. Points hitting artifacts were excluded. Points not hitting cardiomyocytes included interstitial tissue and vasculature. Endocardium was not included. At least 55 points were counted on each section. The relative volume fractions of cardiomyocytes were calculated in percentage as follows:

Cardiomyocytes # Points (Points hitting cardiomyocytes) / # Points (Number of points)

Measurements were made in a blinded fashion.

### Chemicals

Chemical were obtained from Sigma-Aldrich (St. Louis, MO) unless otherwise stated.

### Statistics

Data are presented as means ± SEM. Differences between multiple mean values were analyzed by factorial ANOVA followed by Fisher’s LSD post-hoc test. Comparisons between pairs of mean values were done using Student’s *t*-test. P<0.05 was considered statistically significant. All statistical analyses were performed using STATISTICA 7.0 (StatSoft Inc., Tulsa, OK).

## Results

### General characteristics of the ZDF and ZDL rats

Table 1 shows mean arterial pressure (MAP), body weight and blood triglycerides of the ZDF and ZDL rats. MAP was significantly elevated in ZDF compared to ZDL rats as previously reported [33,34]. Bodyweight was significantly increased in ZDF rats compared to ZDL rats, and ZDF rats were also characterized by hypertriglyceridemia.

**Table 1:** General characteristics of ZDF and ZDL rats

	**ZDF rats (n=16)**	**ZDL rats (n=21)**
Body weight (g)	403 ± 10.1 ***	351 ± 4.3
MAP (mmHg)	121 ± 1.4 ***	108 ± 2.7
Blood triglycerides (mmol/L)	5.0 ± 0.44 ***	1.1 ± 0.08

Mean arterial pressure (MAP), body weight, and blood triglycerides of ZDF and ZDL rats. *** P<0.001 versus ZDL rats by Student’s *t*-test.

### Conduction velocity is reduced in ZDF rats

**Figure 1:**
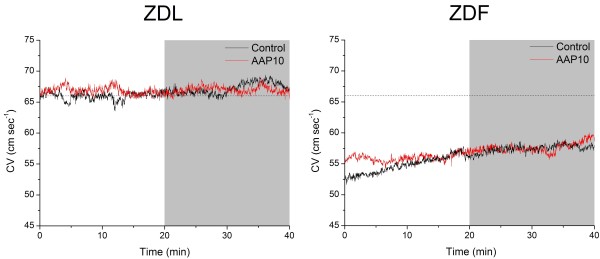
**Conduction velocity.** Conduction velocity (CV) in tissue strips from the right ventricle of ZDL and ZDF rats under control conditions (black lines) or treatment with the anti-arrhythmic peptide AAP10 (50 nmol/L) (red lines). Treatment was started at 20 min as indicated by the gray shaded area in the figure. The dashed line indicates the average CV during the last 10 min of the baseline period in ZDL rats. Mean and SEM values for each group and period are shown in Table 1.

CV was 56±1.9 cm/s at baseline in ZDF rats (n=16), which was significantly slower than CV in ZDL rats (66±1.6 cm/s, n=21, P<0.001) (see Figure 1 and Table 2). AAP10 (50 nmol/L) treatment for 20 minutes did not affect CV in either group. Table 2 also shows the passive and developed force for both untreated and AAP10 treated strips. On average, diastolic force was 349 ± 47.5 mg/mm^2^ in ZDF rats (n=16) and 241 ± 30.8 mg/mm^2^ in ZDL rats (n=21). This difference is borderline significant (P=0.055). Developed force was not different between ZDF and ZDL rats during baseline conditions (ZDF: 268 ± 25.6 mg/mm^2^ and ZDL: 229 ± 16.4 mg/mm^2^, NS). AAP10 had no effect on either diastolic or developed force. However, baseline and treatment values for developed force in the untreated ZDF group were higher than both the ZDL group and the ZDF group which received AAP10 during the experiment. Since treatment was chosen randomly, this difference must have arisen by chance.

**Table 2:** Conduction velocity, diastolic and developed force

	**ZDF**		**ZDL**	
	*Control (n=8)*	*AAP10 (n=8)*	*Control (n=10)*	*AAP10 (n=11)*
**Conduction velocity (cm/s)**				
Baseline	55.9 ± 2.3 #	56.2 ± 3.4 #	66.0 ± 2.9	66.7 ± 1.7
Treatment	57.8 ± 3.2 #	57.9 ± 3.5 #	67.9 ± 2.9	67.0 ± 2.0
**Diastolic force (mg/mm**^**2**^**)**				
Baseline	317 ± 53	380 ± 81	216 ± 44	263 ± 44
Treatment	333 ± 53	386 ± 76	202 ± 38	258 ± 40
**Developed force (mg/mm**^**2**^**)**				
Baseline	331 ± 29 *#	206 ± 29	223 ± 25	234 ± 23
Treatment	330 ± 36 *#	239 ± 33	252 ± 27	271 ± 26

Conduction velocity, diastolic and developed force in control (vehicle treated) and AAP10 treated strips from ZDF and ZDL rats. Data are shown as mean ± SEM during the last 10 min of each period (* P<0.05 for control versus AAP 10; # P<0.05 for ZDF versus ZDL, analyzed by factorial ANOVA followed by Fisher’s LSD post-hoc test).

### Localization and phosphorylation of C×43

**Figure 2:**
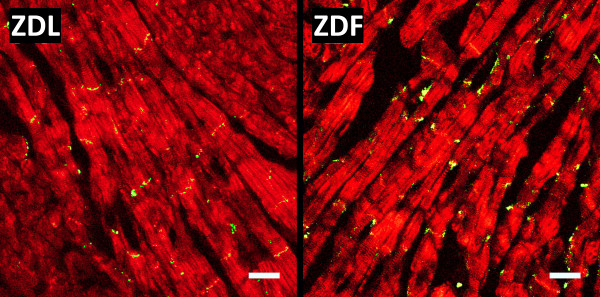
**Connexin43 expression.** Representative staining of Cx43 expression in the right ventricle of ZDL and ZDF rat hearts. Sections of ventricular myocardium were stained for Cx43 (green) and F-actin (red) and used for quantification of total Cx43 levels (Scale bar is 25 μm).

**Figure 3:**
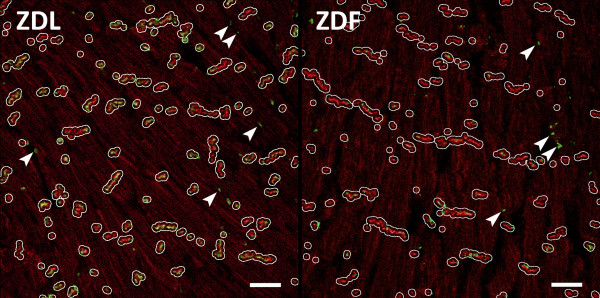
**Connexin43 localization.** Representative images from ZDL and ZDF rats used for quantification of Cx43 colocalizing with N-cadherin. Sections of ventricular myocardium were stained for N-cadherin (red) and Cx43 (green). White lines show the enlarged regions of N-cadherin (see Materials and Methods) used to identify the intercalated disc. Green staining (Cx43) within these regions were regarded as localized in intercalated discs. Cx43 outside the N-cadherin regions was regarded as lateralized. The arrows point to examples of lateralized Cx43. (Scale bar is 25 μm).

Figure 2 shows representative staining of C×43 in right ventricular tissue from a ZDL and a ZDF rat. The total amount of C×43 determined by the summed fluorescence intensity per unit area of myocytes was identical between ZDL and ZDF rats (1.64±0.65 (n=6) versus 1.64±0.62 (n=7), NS). To quantify the localization of C×43 to the intercalated disc co-staining for C×43 and N-cadherin was performed (Figure 3). In ZDL rats 70±8% (n=7) of C×43 was localized at the intercalated discs versus 58±12% (n=7) in ZDF rats. This shows that the amount of lateralized C×43 was increased in the diabetic rats (P<0.04).

Western blotting was used to measure overall C×43 phosphorylation. In the present study we dephosphorylated rat ventricular protein and included it as a standard on all gels to identify the localization of the C×43 P0 band. This allowed us to define the ratios between P0 and higher phosphorylated forms of C×43.

In tissue taken from the right ventricle of ZDL rats 47 ± 5.2% (n=15) of the total C×43 migrated in the P0 form, which was not significantly different from ZDF rats (50.7 ± 5.6%, n=18) (data not shown). This indicates that the overall degree of C×43 phosphorylation is fairly identical between ZDF and ZDL rats. However, it does not exclude that some site specific phosphorylation changes have occurred.

### Cardiomyocyte size is unchanged between ZDF and ZDL rats

Cell dimensions were measured on longitudinally sectioned myocytes with clear boundaries and visible intercalated discs. The measurements were performed on the images used for quantification of C×43 lateralization (Figure 3). Average cell length was 64 ± 7.4 μm in ZDF rats (102 cells from 8 rats), which was not significantly different compared to the ZDL rats (60 ± 19.7 μm, 92 cells from 7 rats, NS). Likewise, measurement of cross sectional area of the myocytes showed no difference between the two groups (992 ± 50.4 μm^2^ in ZDF versus 982 ± 53.9 μm^2^ in ZDL, NS).

### Cardiac histology is unchanged in the ZDF rats

**Figure 4:**
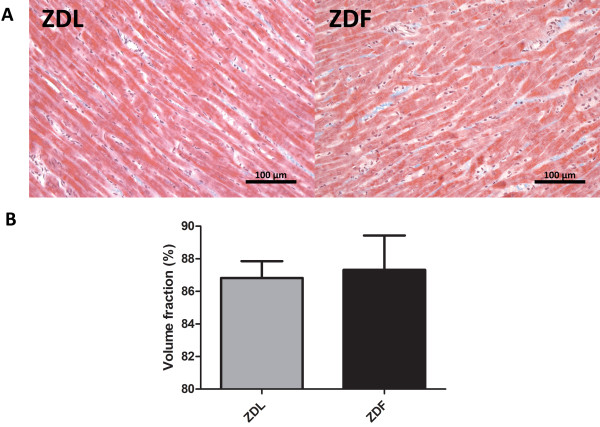
**Cardiac histomorphometry. A)** Representative images of the Masson`s Trichrome staining of right ventricular tissue from ZDL and ZDF rats. **B)** The mean volume fraction of healthy cardiomyocytes (n = 5 per group and data are shown as mean ± SEM).

Representative Masson`s Trichrome stainings from ZDF og ZDL rat hearts are shown in Figure 4. Tissue samples from neither ZDF nor ZDL rats showed any signs of histomorphometric changes in the form of collagen or lipid infiltration and the volume fraction of healthy cardiomyocytes was identical between the two groups.

### ZDF rats have intramyocardial lipid accumulation

**Figure 5:**
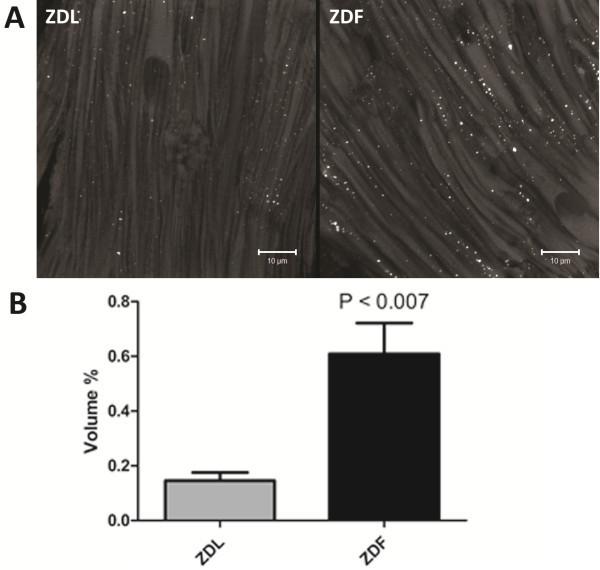
**Intramyocardial lipid droplets. A)** Representative image of the intracellular distribution of lipid droplets stained with Bodipy-493/503 (white spots) in right ventricular tissue from ZDL and ZDF rats. **B)** The mean relative volume occupied by lipid (n = 4 per group and data are shown as mean ± SEM).

As shown in Figure 5, tissue from the right ventricle of ZDF rats show significantly increased intramyocardial lipid accumulation compared to tissue from ZDL rats. Our staining showed a significant increase in the number of lipid droplets pr. μm^3^ (0.13 ± 0.02 vs. 0.07 ± 0.01, P<0.014) as well as in the actual size of each lipid droplet (0.05 ± 0.009 μm^3^ vs. 0.02 ± 0.003 μm^3^, p< 0.020) in ventricular tissue form ZDF compared to ZDL rats. On average, the total intramyocardial lipid volume fraction was 4.2 times higher in ZDF compared to ZDL rats (Figure 5B).

## Discussion

Here we show that propagation of the electrical impulse is significantly slowed in the right ventricle of ZDF rats compared to ZDL rats. To our knowledge, this is the first study to report decreased cardiac CV in an animal model of type 2 diabetes.

Our finding is in accordance with studies performed in STZ induced type 1 diabetic Wistar rats, which show decreased CV at unstressed conditions [17] and a decreased conduction reserve [17-19,35]. In addition, QRS prolongation, which may occur due to decreased CV, is found in both STZ treated rats [36] and ZDF rats [27], as well as in diabetic humans [37]. This indicates that decreased CV may be an independent risk factor for development of ventricular arrhythmias in both type 1 and 2 diabetic patients.

CV slowing can occur by either reduced excitability, increased internal electrical resistance or by obstacles to conduction such as fibrosis [38]. CV is normally very stable presumably due to the existence of a conduction reserve and therefore large alterations are needed to slow CV (for review see [8]). The internal electrical resistance is determined by both the electrical resistance of the cell interior and by the gap junction mediated coupling between the cells. Previous studies on rats with STZ induced type 1 diabetes indicate changes in expression, localization, and phosphorylation of the major ventricular gap junction protein C×43, but the reports are contradicting. Some studies report unchanged levels of C×43 [19,20], whereas other report reduced [17,18] or increased levels [20,21]. The discrepancies between studies may be design dependent; Lin et al. showed that C×43 levels were increased 5 weeks after STZ treatment, but C×43 levels were back to normal after 10 weeks [20]. In this study, we found no difference in the level of C×43 between ZDF and ZDL rats, but we did see a statistically significant, increase in C×43 lateralization, measured as the fraction of C×43 not localized in close proximity of N-cadherin, in ZDF rat hearts. Lateralization of C×43 is frequently reported in cardiac diseases, and it is often assumed that lateralized C×43 is nonfunctional. When analyzing C×43 localization based on the shape of the cardiomyocytes, it should, however, be noted that gap junctions, which are located at the “long side” as opposed to the “end” of the cell (where the endplates are located), may none the less still be located in intercalated discs, and thus be functional. In this regard, it is of interest to note that one study has found that lateralization of C×43 improves lateral coupling [39], which indicates that lateralized gap junctions may not be nonfunctional. Furthermore, it was previously shown that interaction between connexins and the N-cadherin-catenin complex found in intercalated discs, is required to maintain gap junction functionality [40]. Therefore, we chose to evaluate gap junction lateralization as the fraction of C×43 that did not appear in close proximity of N-cadherin. The observed increase in lateralization of C×43 in ZDF rats may account for part of the observed decrease in CV. Studies in heterozygous C×43 knockout mice have shown contradicting effects on CV following a 50% reduction in C×43 expression; some studies report a decreased CV [41-43], whereas other studies did not detect significant decreases in CV [44,45]. Based on the above, we find it unlikely that the 17% decrease (from 70 to 58%) in C×43 found in the intercalated discs solely accounts for the observed decrease in CV. In contrast to C×43 lateralization, it is well documented that C×43 phosphorylation plays an important role in gap junction coupling [46,47] and dephosphorylation of C×43 has been related to electrical uncoupling in ischemia [48,49]. Judged by the electrophoretic mobility of C×43, we did not observe any differences in overall C×43 phosphorylation between ZDF and ZDL rat hearts. This is in contrast to studies on STZ rats where mobility shifts indicates hyperphosphorylation [17,20,21]. However, since more than twenty phosphorylation sites are present in C×43, we cannot exclude that some changes in C×43 phosphorylation may have occurred in the ZDF rats and a complete phosphorylation analysis may be an interesting subject for future work. Nonetheless, our data indicate that changes in C×43 expression, localization or phosphorylation cannot by itself explain the observed decrease in CV in ZDF rats. This interpretation is further supported by the fact that AAP10 did not affect CV in the ZDF rats. Previous studies have shown that the AAP analogue, rotigaptide, prevents electrical uncoupling [50] and CV slowing during ischemic conditions with no effect under unstressed conditions [51] and that it also effectively reverts established CV slowing [52]. AAP10 and its analogs are believed to affect gap junction coupling through changes in C×43 phosphorylation [48], which is a rapid process occurring within minutes. Therefore, the lack of an AAP10 effect in the ZDF hearts implies that the mechanism of CV slowing in diabetes is different from that seen in ischemia, where gap junction uncoupling by C×43 dephosphorylation is a major contributor.

In addition to gap junction coupling, cell size is also important for CV because the cell dimensions determine both how many high resistance barriers (cell-cell junctions) the impulse has to cross per unit length and the cross sectional area of the cell available for conducting current. Cell size is reduced in STZ induced diabetes due to removal of the hypertrophic effect of insulin [19]. Type 2 diabetes on the other hand is characterized by initial hyperinsulinemia that together with the increase in blood pressure in ZDF rats (Table 1) may lead to hypertrophy (for review see [53]). In the present study, we found no change in cell dimensions, ruling out that atrophy or hypertrophy causes the observed changes in the propagation of the electrical impulse.

A mild increase in cardiac fibrosis has previously been reported for ZDF rats at both 7, 14 and 21 weeks of age [26]. Fibrosis may compose an obstacle to electrical conduction and thereby contribute to a decrease in conduction velocity. Our histological analysis, however, showed absolutely no histomorphological differences between ZDF and ZDL rats.

Since the CV slowing we observe in ZDF rats does not seem to be fully explained by changes in either gap junction remodeling, changes in cell size or other morphological changes, we hypothesize that other factors are involved in the development of conduction disturbances in type 2 diabetes. Lipotoxicity due to ectopic lipid accumulation in the heart is a well known phenomenon in diabetes. Lipotoxicity has mainly been connected to impairment of cardiac energy metabolism and lipoapoptosis (for recent review see [54]), but a number of studies suggests that altered energy metabolism may compromise the function of cardiac ion channels (for review see [55]). Our lipid staining revealed significant intramyocardial lipid accumulation in ZDF compared to ZDL rats, which has also previously been shown by chloroform-methanol extraction [25,26]. Therefore, we hypothesize that intramyocardial lipid accumulation and/or altered lipid metabolism may be involved in development of conduction disturbances in ZDF rats. Previous studies have shown that the fatty acid metabolite palmitoylcarnitine causes a concentration dependent decrease in the transient outward (*I*_to_) K^+^ current [56], as well as the Na^+^ current [57] in isolated cardiomyocytes. In addition, mice with cardiac-specific over expression of the peroxisome proliferator-activated receptor α (PPARα) (a key player in the regulation of cardiac lipid metabolism) also displays decreased *I*_to_ density [58]. Changes in the *I*_to_ current are not directly related to conduction disturbances, but decreased Na^+^ current density reduces cardiomyocytes excitability and may thereby slow conduction. Diabetes also correlates to reduced function of the sarcolemmal Na^+^/K^+^ pump, due to changes in ATP levels [59]. Altered Na^+^/K^+^ pump function may lead to intracellular Na^+^ accumulation and K^+^ depletion, which may depolarize the resting membrane potential and increase the fraction of inactivated Na^+^ channels. A combination of reduced Na^+^ channel density and increased fraction of inactivated channels could contribute to the decreased CV observed in ZDF rats. It does, however, remain an important question for future research whether the metabolic alterations are sufficient to cause altered ion channel function and/or disturbances in the trans-membrane ion gradients, which is of a magnitude sufficient to explain the observed decrease in CV.

## Conclusions

CV is reduced in the right ventricle of ZDF rats, which indicates that decreased CV may be an independent risk factor for development of ventricular arrhythmias in type 2 diabetic patients. Despite a small degree of C×43 lateralization the conduction disturbance does not seem to be mediated solely by altered gap junction coupling. Instead, the role of increased intracellular lipid and its effect on cardiomyocyte excitability is an interesting subject for future research.

## Abbreviations

AAP: Anti-arrhythmic peptide; CV: Conduction velocity; C×43: Connexin 43; ECG: Electrocardiogram; MAP: Mean arterial blood pressure; NS: Non significant; PIP2: Phosphatidylinositol-bisphosphate 2; PPARα: Peroxisome proliferator-activated receptor α; STZ: Streptozotocin; ZDF: Zucker diabetic fatty; ZDL: Zucker diabetic lean.

## Competing interests

The authors declare that they have no conflict of interests.

## Authors’ contributions

KBO participated in the design of the study, conducted CV measurements, immune- and Masson`s Trichrome staining, as well as western blots and she was involved in the data analysis and drafting of the manuscript. LNA participated in the design of the study, lipid staining, data analysis, statistics and drafting of the manuscript. THB carried out lipid- and immunostainings, data analysis and was involved with the drafting of the manuscript. CMS participated in blood pressure measurements and critical review of the manuscript. CBA carried out Masson`s Trichrome staining, data analysis thereof and critical review of the manuscript. TP was involved in the lipid staining and critical review of the manuscript. NHHR participated in the design of the study, wrote the costume written MatLab program used for data analysis, conducted statistical analysis and critical review of the manuscript. MSN participated in the design of the study, lipid staining, statistical analysis and drafting of the manuscript. All authors have read and approved the final manuscript.
